# Facile Synthesis of Reduced-Graphene-Oxide-Modified Ammonium Polyphosphate to Enhance the Flame Retardancy, Smoke Release Suppression, and Mechanical Properties of Epoxy Resin

**DOI:** 10.3390/polym15051304

**Published:** 2023-03-05

**Authors:** Feiyue Wang, Jiahao Liao, Miaotian Long, Long Yan, Mengtao Cai

**Affiliations:** Institute of Disaster Prevention Science and Safety Technology, School of Civil Engineering, Central South University, Changsha 410075, China

**Keywords:** epoxy resin, ammonium polyphosphate, graphene oxide, flame retardancy, char formation

## Abstract

A unique hybridized intumescent flame retardant named reduced-graphene-oxide-modified ammonium polyphosphate (RGO-APP) was successfully synthesized via the simple hydrothermal method and reduced process. Then, the obtained RGO-APP was applied in epoxy resin (EP) for flame retardancy reinforcement. The addition of RGO-APP results in a significant reduction in heat release and smoke production from the EP, which is attributed to EP/RGO-APP producing a more compact and intumescent char against the heat transfer and combustible decomposition, thus enhancing the fire safety of EP, as confirmed by char residue analysis. Especially, the EP containing 15 wt% RGO-APP acquires a limiting oxygen index (LOI) value of 35.8% and shows a 83.6% reduction in peak heat release rate and a 74.3% reduction in peak smoke production rate compared with those of pure EP. The tensile test exhibits that the presence of RGO-APP favors the enhancement in tensile strength and elastic modulus of EP due to the good compatibility between flame retardant and epoxy matrix, as supported by differential scanning calorimetry (DSC) and scanning electron microscope (SEM) analyses. This work provides a new strategy for the modification of APP, thus facilitating a promising application in polymeric materials.

## 1. Introduction

Epoxy resin (EP) is widely used in mechanical manufacturing, aerospace, electronics, electrical products, chemical anticorrosion, and other fields thanks to its good adhesion, corrosion resistance, high hardness, and superior mechanical properties [1]. However, the flammability of epoxy resin severely limits its application in semiconductor packaging and circuit printing plates [2,3]. Obviously, it is necessary to modify epoxy resin with various flame retardants. With the improvement in people’s requirements for environmental safety and health levels, halogen-free flame retardants are gradually replacing halogen-containing flame retardants as the new trend in epoxy resin flame retardants [4,5]. Ammonium polyphosphate (APP) is a common halogen-free phosphorus flame retardant, which has the advantages of environmental protection, low cost, and good flame retardancy [6,7]. However, APP cannot meet the corresponding requirements in the practical application of flame-retardant-modified polymers owing to its poor compatibility [8,9]. In addition, APP, as an intumescent flame retardant, is generally only used as an acid source and a gas source, and cannot be used as a carbon source. Therefore, the combination of APP and a carbon source can usually achieve better flame-retardant performance [10,11].

Graphene is a kind of two-dimensional honeycomb lattice material with tightly packed carbon atoms, which can act as a physical barrier to delay heat transfer and release during combustion [12,13,14]. However, the dispersion of untreated graphene in the polymer matrix is poor and the stability of the char layer formed in combustion is not satisfactory [15,16,17,18,19,20]. The weak interaction with the substrate also leads to the weakening of mechanical properties. In addition, it is difficult to show satisfactory flame retardancy when graphene is used as flame retardant alone [21,22]. Therefore, the combination of graphene and a traditional flame retardant such as APP has attracted the interest of researchers [23,24]. Shi [25] mixed graphene with ammonium polyphosphate modified by ethylene trimethyl silane, and added it to polystyrene to prepare flame-retardant polystyrene composites. The results showed that the graphene and the ammonium polyphosphate modified by silane had a cooperative effect in improving the thermal stability and flame-retardant properties of polystyrene. Chen [26] studied the cooperative flame-retardant effect of iron-modified graphene and ammonium polyphosphate in thermoplastic polyurethane. The results showed that, when the mass of graphene was 0.25 wt% and the mass of ammonium polyphosphate was 9.75 wt%, the cooperative effect was the strongest, which could increase the limiting oxygen index (LOI) of polyurethane to 38.3% and reduce the heat release rate (HRR) and peak smoke production rate (PSPR) by 92.8% and 72.8%, respectively. Wang [27] prepared graphene-ammonium polyphosphate aerogel (GAPPA) using graphene and ammonium polyphosphate as the main raw materials and filled it with polymethyl methacrylate (PMMA) to synthesize PMMA/GAPPA composites. The results showed that, compared with PMMA, the total heat release (THR) and peak heat release rate (PHRR) of PMMA/GAPPA decreased by 58.7% and 67.2%, respectively, accompanied by excellent mechanical properties. Han [28] studied the flame-retardant and thermal stability of graphene oxide with different oxidation degrees in polymers. The results showed that graphene oxide with lower oxygen functional groups can lead to polymers with stronger thermal stability and compatibility.

In summary, it can be seen that the addition of graphene or APP can effectively improve the flame retardancy of polymers, but there are few relevant studies on using graphene oxide (GO) as a carbon source and APP as both an acid source and a gas source to fabricate a novel hybridized intumescent flame retardant. In this study, reduced graphene oxide (RGO) was selected as the modified material of APP to obtain RGO-APP. The effects of RGO-APP on flame retardancy and thermal stability of EP were evaluated by LOI, UL94 test, and TG analysis. The morphology and chemical structure of char residues were characterized by scanning electron microscopy (SEM) and Fourier transform infrared spectroscopy (FTIR), and the potential flame-retardant mechanism of RGO-APP on EP was studied.

## 2. Experimental

### 2.1. Materials

Ammonium polyphosphate (form II) was supplied by Hangzhou JLS Flame Retardants Chemical Co., Ltd. (Hangzhou, China). Graphite oxide (mass fraction of C element: 47 ± 5%, molar ratio of C/O: 0.6 ± 0.1) was purchased from Sixth Element Materials Technology Co., Ltd. (Changzhou, China). L-ascorbic acid (LAA) was purchased from Shanghai Aladdin Biochemical Technology Co., Ltd. (Shanghai, China). Epoxy resin (E-44) was obtained from Zhenjiang Danbao Resin Co., Ltd. (Zhenjiang, China). 4,4-Diaminodi-phenylmethane (DDM) was purchased from Changzhou Runxiang Chemical Co., Ltd. (Changzhou, China). 3-aminopropyltriethoxysilane (KH550) was obtained from Jiangsu Chenguang Coupling Reagent Co., Ltd. (Zhenjiang, China). Ethanol was supplied by Tianjin Hengxing Chemical Reagent Manufacturing Co., Ltd. (Tianjin, China). All of the reagents are used as received.

### 2.2. Preparation of RGO-APP Flame Retardant

**Figure 1:**
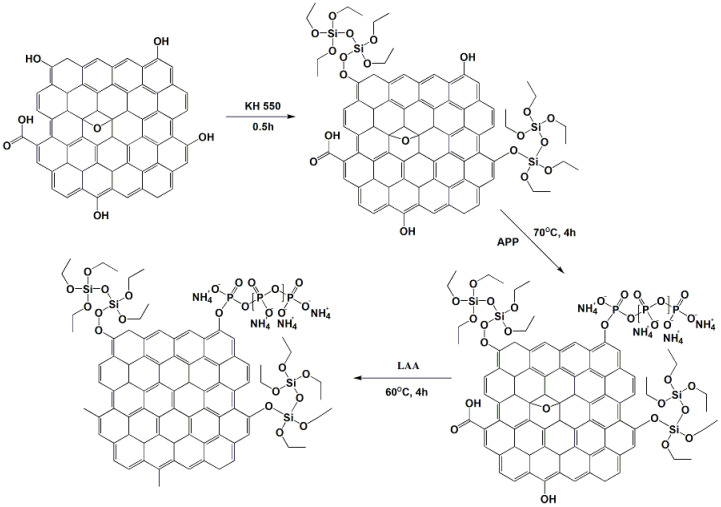
Synthesis route of RGO-APP flame retardant.

Firstly, 2 g of graphite oxide powder and 400 mL of solution (ethanol/water = 1:1) were fully mixed and ultrasonically dispersed for 1 h. Then, 0.1 g of KH550 was added into the above solution with stirring for 0.5 h to obtain a graphene oxide solution with good dispersion. After that, APP (10 g) was added into the above solution, and the solution was injected into a 500 mL three-necked flask and heated at 70 °C for 4 h. To remove the excess oxygen-containing groups from the GO surface, 12 g LAA was added to the above solution and it was stirred at 60 °C for 4 h. The specific formulation is shown in Table 1 and the synthetic route of RGO-APP is shown in Figure 1.

RGO was prepared by the ultrasonic dispersion method and hydrothermal reduction method. Firstly, a graphene oxide solution with good dispersion was prepared. Then, 12 g of LAA solution was injected into the graphene solution and hydrothermally reduced at 60 °C for 4 h. The obtained solution was filtered, washed three times with ethanol solution, and dried in an oven to a constant weight to obtain RGO. Moreover, RGO@APP was obtained by physically blending RGO and APP with a mass ratio of 1:6.

### 2.3. Preparation of EP and EP Composites

Firstly, EP was preheated to 60 °C for softening and DDM was heated to a molten state in a 90 °C water bath. An appropriate amount of EP was mixed with a quantitative flame retardant under stirring at 400 r/min for 10 min at 80 °C. Then, the temperature was adjusted to 90 °C and the molten DDM (EP/DDM = 4:1) was added to the above mixture, and the mixture was stirred at 400 r/min for 2 min. The above EP mixture was quickly poured into the preheated polytetrafluoroethylene (PTFE) mold and placed in an electric heating blast drying oven. The pre-curing temperature was 120 °C for 2 h, and the curing temperature was 150 °C for 1 h. After demolding and cooling to room temperature, the corresponding flame-retarded EP was obtained.

### 2.4. Characterization and Measurement

Fourier transform infrared (FTIR) spectra were obtained on an I CAN 9 infrared spectrometer (Tianjin Energy Spectrum Technology Co., Ltd., Tianjin, China) in a wavenumber range of 4000–500 cm^−1^.

Scanning electronic microscopy (SEM) was utilized to obtain the microcosmic morphologies of the samples at an accelerating voltage of 20 kV on a TESCAN MIRA3 LMU (TESCAN CHINA, Ltd., Brno, Czech Republic). Energy-dispersive spectroscopy (EDS) was equipped to study element analysis on an X-Max20 X-ray probe (Oxford Instruments Co., Ltd., Abingdon Oxon, UK).

Thermogravimetry (TG) analysis was obtained on a TGA/SD-TA851e thermal gravimetric analyzer (Mettler Toledo International Trading Co., Ltd., Shanghai, China). The sample (about 5 mg) was heated from 25 to 800 ℃ under an air atmosphere of 50 mL/min. The theoretical char residue (*W_cal_*) was calculated according to Formula (1).

(1) $$W_{cal}\left(T\right)=\overset{n}{\underset{i=1}{\sum }}x_{i}w_{i}\left(T\right)\;\;\;$$

where $x_{i}$ is the content of compound *i* and $w_{i}$ is the experimental weight of compound *i*.

Differential scanning calorimetry (DSC) analysis was performed using DSC823e (Mettler Toledo International Trading Co., Ltd., Shanghai, China). The sample was heated to 200 °C and maintained for 3 min, and then cooled down to room temperature at a cooling rate of 10 °C/min to eliminate the thermal history. Then, the sample was heated at a heating rate of 10 °C/min from 25 °C to 200 °C under a nitrogen atmosphere of 50 mL/min.

The limiting oxygen index (LOI) value of pure EP and EP composites was obtained using an HC-2CZ oxygen index meter (Nanjing Shangyuan Analytical Instrument Co. Ltd., Nanjing, China) according to ASTM D2863-19 “Standard test method for measuring the minimum oxygen concentration to support candle-like combustion of plastics (oxygen index)” with a sample size of 130 × 6.5 × 3.2 mm^3^.

UL94 test was conducted using a JL8333-3 horizontal combustion tester (Nanjing Jionglei Instrument Equipment Co., Ltd., Nanjing, China). The test is according to the procedure of UL94-2016 “Standard for safety: Tests for flammability of plastic materials for parts in devices and appliances”, and the specimens for the test are 130 × 13 × 3.2 mm^3^.

A cone calorimeter (FTT 0007) was utilized to investigate the combustion behavior of EP composites according to ISO 5660-1:2015 “Reaction-to-fire tests-Heat release, smoke production, and mass loss rate-Part 1: Heat release rate (cone calorimeter method) and smoke production rate (dynamic measurement)”. All of the samples for testing were horizontally irradiated at a heat flux of 50 kW/m^2^ with a size of 100 × 100 × 3.5 mm^3^.

The mechanical test was performed in a WDW-10D microcomputer-controlled electronic universal testing machine (Jinan Hengsi shengda Instrument Co., Ltd., Jinan, China) according to GB/T1040.2-2006 “Plastics-Determination of tensile properties-Part 2: Test conditions for molding and extrusion plastics”. The sample size is the type I standard sample and the test speed is 5 mm/min.

## 3. Results and Discussion

### 3.1. Characterization of RGO-APP Flame Retardant

**Figure 2:**
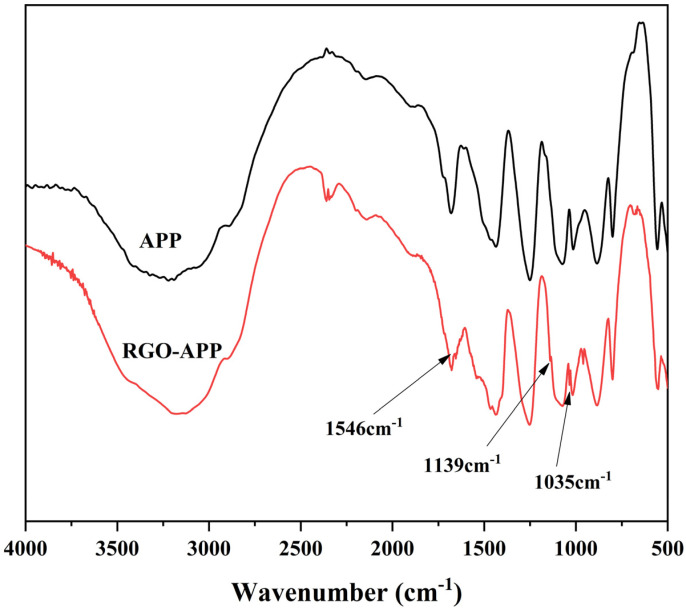
FTIR of APP and RGO-APP.

Figure 2 presents the FTIR spectra of APP and RGO-APP. Compared with APP, the intensity of the characteristic peak of RGO-APP at 1259 cm^−1^ is slightly reduced, which is attributed to the covering effect of the loaded RGO on the P=O bond on the APP surface. In addition, two new characteristic peaks appeared at 1546 cm^−1^ and 1139 cm^−1^ in the RGO-APP spectrum, caused by the skeleton vibration of graphene and the Si–O–Si and Si–O–C stretching vibration, respectively, indicating that KH560 is successfully introduced onto the surface of RGO [29,30,31]. In addition, a new peak appeared at 1035 cm^−1^, attributed to the P-O-C bond formed by the reaction of APP and RGO, indicating the formation of the chemical bond between RGO and APP [32].

**Figure 3:**
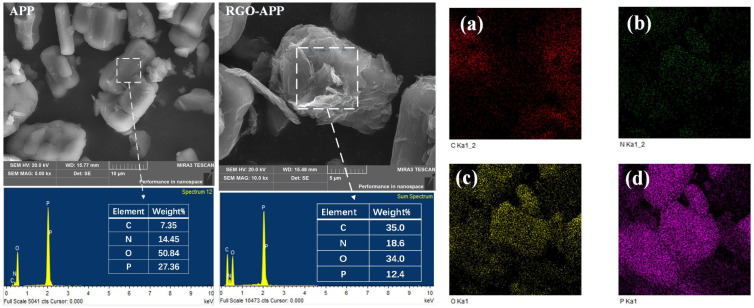
SEM images and EDS maps of APP and RGO-APP and element distribution of RGO-APP: (**a**) Carbon element, (**b**) Nitrogen element, (**c**) Oxygen element, (**d**) Phosphorus element.

The SEM images and EDS maps of APP and RGO-APP are shown in Figure 3. It can be seen from the SEM images that the original smooth APP surface becomes rough owing to the grafting of flake structures, indicating that the graphene has successfully adhered to the surface of APP. It can be seen from the EDS spectrum that the carbon element on the RGO-APP surface increases significantly. Moreover, the oxygen and phosphorus elements on the RGO-APP surface are decreased owing to the covering effect. The above results indicate that RGO-APP is successfully prepared [33,34].

### 3.2. TG Analysis

**Figure 4:**
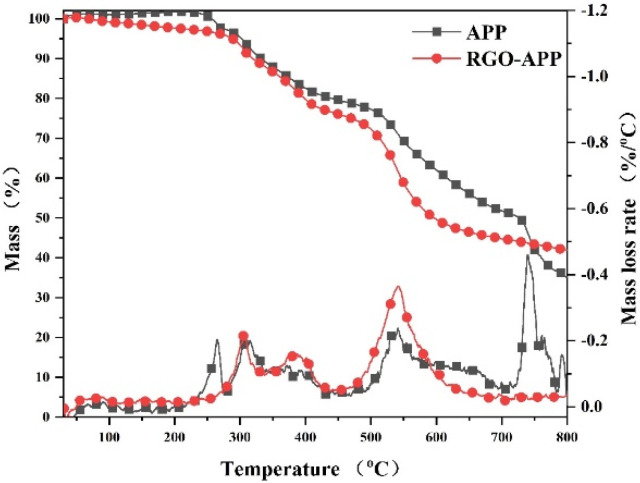
Thermogravimetric test results of APP and RGO-APP.

The thermal decomposition process of APP and RGO-APP at 25–800 °C in a nitrogen atmosphere is shown in Figure 4. As shown in the diagram, both APP and RGO-APP present four decomposition stages in the temperature ranges of 200–300 °C, 300–500 °C, 500–600 °C, and 600–800 °C, respectively. Compared with APP, RGO-APP has a weaker thermal stability between 600 and 800 °C, accompanied by a higher residual weight. Especially, the residual weights of APP and RGO-APP at 800 °C are 35.5% and 42.0%, respectively. The above results show that the thermal decomposition process of APP has changed significantly after grafting RGO, and the char-forming ability is significantly improved.

**Figure 5:**
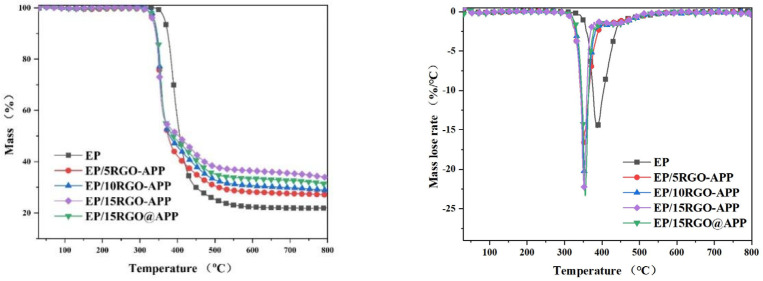
TG and DTG curves of pure EP and flame-retardant EP.

The TG and DTG curves of pure EP and flame-retarded EP with RGO-APP under a nitrogen atmosphere are shown in Figure 5, and the relevant thermal analysis data are shown in Table 2.

It can be seen from Figure 5 that all of the samples show an obvious pyrolysis process at 300–500 °C. The addition of flame retardants significantly decreases the initial thermal decomposition temperature (T_5%_) of EP owing to the earlier decomposition of flame retardants. After adding RGO-APP, the maximum thermal decomposition temperature (T_max_) of EP increases gradually, which is consistent with the changing trend of T_5%_. In addition, it can be found that the peak mass loss rate (PMLR) increases with the increase in RGO-APP content, which is caused by the decomposition of APP at a low temperature. Moreover, the residual weight (W_exp_) of EP increases significantly with the increase in RGO-APP content, where the EP composite containing 15 wt% RGO-APP shows the highest residual weight at 800 °C of 33.8%. Compared with EP/15RGO@APP, it can be found that the T_5%_ and T_max_ of EP/15RGO-APP are higher, indicating that the thermal barrier effect of RGO-APP is stronger than that of RGO@APP.

### 3.3. DSC Test

**Figure 6:**
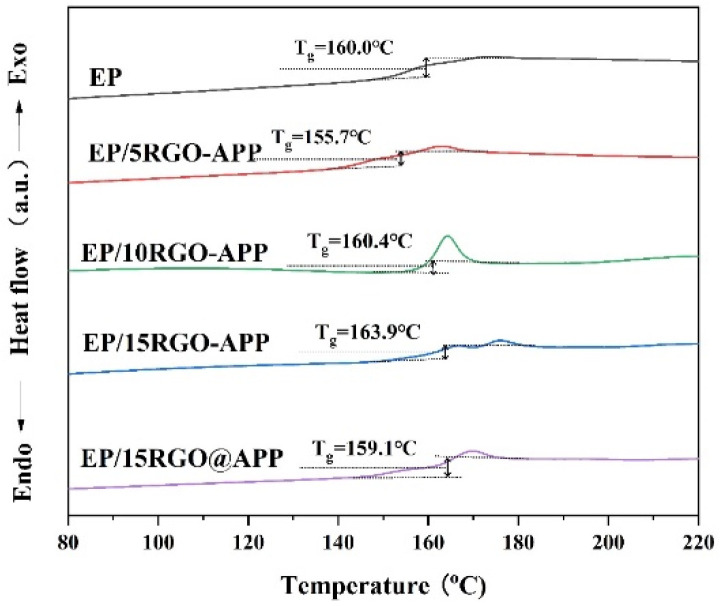
Differential scanning calorimeter (DSC) curves of pure EP and flame-retardant EP.

In order to study the effect of RGO-APP on the glass transition temperature (T_g_) of EP, differential scanning calorimetry (DSC) analysis was carried out. The DSC curves of EP are shown in Figure 6. The T_g_ of pure EP is 160.0 °C and the addition of 5 wt% RGO-APP can reduce the glass transition temperature of EP. However, the T_g_ of EP gradually increase when the addition amount is higher than 10 wt%. The above situation is because the interaction between APP on the surface of RGO-APP and EP is weak at a low addition level. Notably, the T_g_ value of EP increases with the further increase in RGO-APP content, which is due to the strong interaction of RGO caused by the increase in the specific surface area, thus limiting the movement of polymer molecular chains [35]. Especially, EP/15RGO-APP has a higher T_g_ value than that of EP/15RGO@APP, which is attracted to the good cross-linked network compatibility and interface adhesion between RGO-APP and the EP matrix.

### 3.4. LOI and UL94 Tests

**Figure 7:**
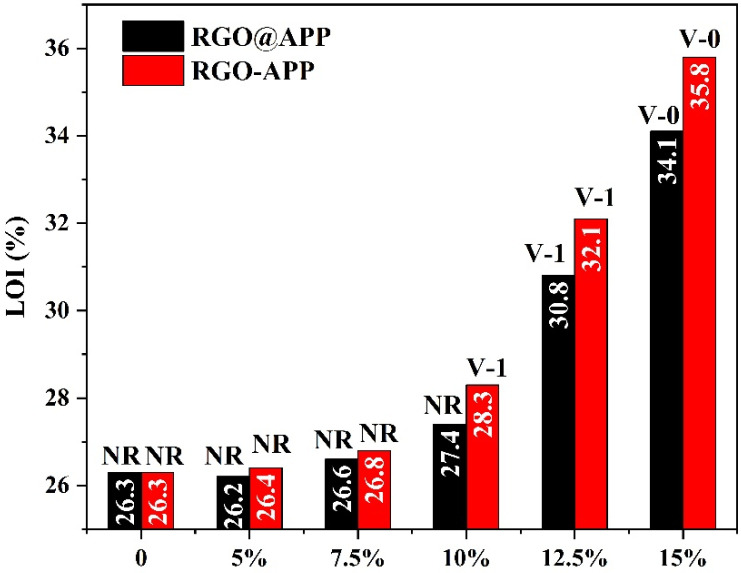
LOI values and UL94 ratings of flame-retardant EP.

The flame-retardant performance of RGO-APP in EP was evaluated by the limiting oxygen index (LOI) test and UL94 test. The results are shown in Figure 7 and Table 1. The LOI value of pure EP is only 26.3% and fails to pass the UL94 test. When the additional amount of RGO@APP is less than 10 wt%, the LOI value and UL94 grade of EP are not significantly improved, while 10 wt% RGO-APP endows EP with excellent flame retardancy accompanied by an LOI value of 28.3% and UL94 V-1 rating. After adding 15 wt% of flame retardant, the sample containing RGO-APP has the highest LOI value of 35.8%, while that of the sample containing RGO@APP is only 34.1%, further indicating the higher flame-retardant efficiency of RGO-APP than that of RGO@APP.

### 3.5. Cone Calorimeter Test

**Figure 8:**
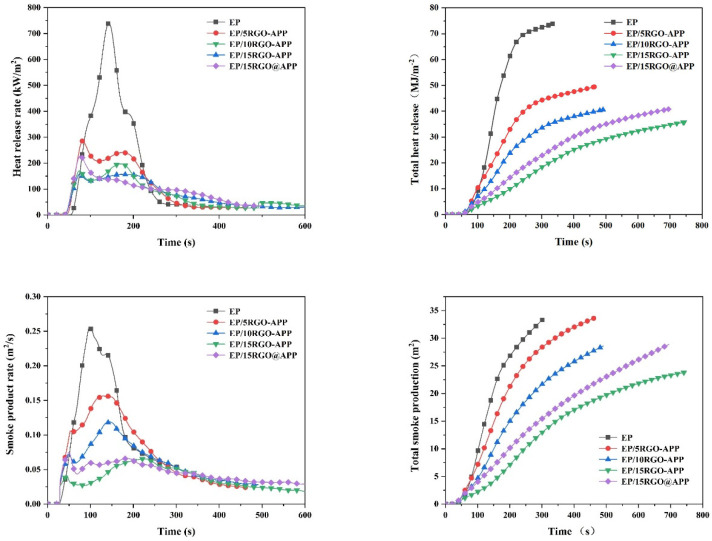
HRR, THR, SPR, and TSP curves of pure EP and flame-retardant EP.

In order to further understand the inhibition of RGO-APP on the heat release and smoke production of EP, the cone calorimeter test was carried out. The results are shown in Figure 8 and the specific values are shown in Table 3. It can be seen that both RGO-APP and RGO@APP have an obvious inhibitory effect on the heat release of EP. From the perspective of time to ignition (TTI) value, the addition of RGO-APP can delay the ignition process of EP at high temperatures, and the TTI value of EP/15RGO-APP is greater than that of EP/15RGO@APP. Moreover, the addition of RGO-APP can greatly reduce the PHRR value of EP. EP/15RGO-APP shows a PHRR value of 157.7 kW/m^2^, while the sample containing 15 wt% RGO@APP has a PHRR value of 227.7 kW/m^2^. From the THR curves, the THR values of EP/15RGO-APP and EP/15RGO@APP are 35.8 MJ/m^2^ and 40.8 MJ/m^2^, respectively, which are 62.6% and 57.4% lower, respectively, than those of pure EP. The above results further show that RGO-APP has a stronger inhibitory effect on heat release in EP than RGO@APP.

From the SPR and TSP curves, the addition of RGO-APP has a significant inhibitory effect on the smoke release of EP. With the increase in RGO-APP content, the PSPR value of EP decreases rapidly. Especially, EP/15RGO-APP shows the lowest PSPR value of 0.0653 m^2^/s and the lowest total smoke release (TSR) value of 23.8 m^2^/m^2^, which are 74.3% and 28.7% lower, respectively, than those of pure EP, exhibiting a super smoke suppression effect. Moreover, it is noted that the TSR value of EP/5RGO-APP is slightly greater than that of pure EP. The above experimental phenomenon reveals that the graphene at a low content has a weak barrier effect on heat and combustible gas, which enables the graphene to be easily brought into the air to form smoke particles, resulting in an increase in smoke production [36,37,38,39,40,41]. With the increased loading of flame retardant, both RGO-APP and RGO@APP show an obvious smoke suppression effect, which is ascribed to the generation of more intumescent char against the release of combustible gases and smoke particles. In general, the formation of intumescent char has a positive effect on inhibiting the heat release and smoke release of EP. Especially, EP/15RGO-APP has the highest char residue of 13.1% among the samples, accompanied by the lowest smoke production and heat release rate.

### 3.6. Char Residue Analysis

**Figure 9:**
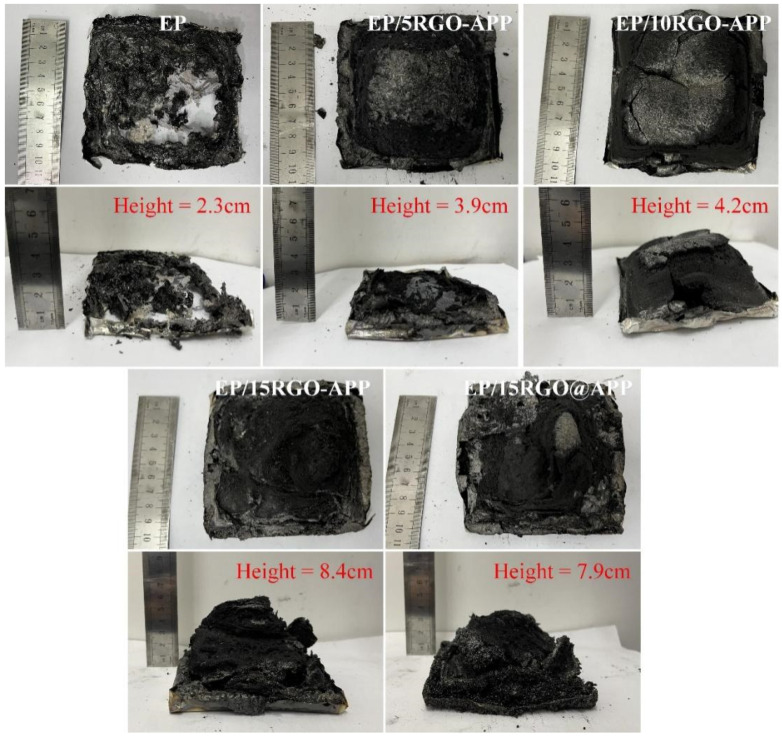
The top view and side view of the char layer of pure EP and flame-retardant EP after the cone test.

The top view and side view of the char layer of EP composites after combustion are shown in Figure 9. The char height of pure EP after combustion is only 2.3 cm, and the char structure is broken and discontinuous. After adding RGO-APP, the char height of EP composites gradually increases with the increase in RGO-APP content, and the macroscopic morphology of the char layer changes from a fragmented structure to a continuous and complete structure. Especially, the char height of the EP/15RGO-APP reaches 8.4 cm, which is 0.5 cm higher than that of EP/15RGO@APP, indicating that RGO-APP has stronger char-forming ability.

**Figure 10:**
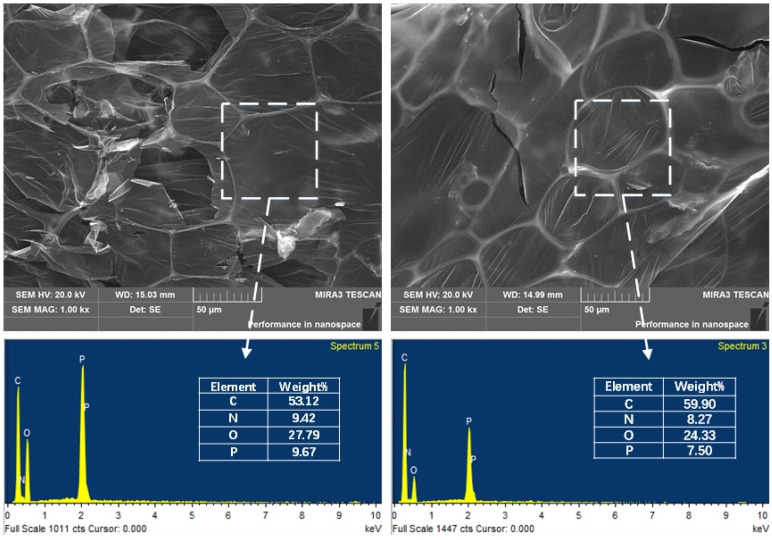
SEM images and EDS maps of the char residues of EP/15RGO@APP (**left**) and EP/15RGO-APP (**right**) were obtained after the cone calorimeter test.

Figure 10 shows the SEM images and EDS maps of the char layer of EP samples after the cone calorimeter test. The char of EP/15RGO@APP has many holes and square bubbles, while EP/15RGO-APP char has only a few cracks and elliptical bubbles on the surface. Generally, the char layer with elliptical bubbles helps to form the “twisted channel” of heat and combustible gas transmission. RGO-APP has a stronger char-forming ability and flame-retardant effect than RGO@APP, which can be ascribed to the fact that the high specific surface area of RGO-APP provides a carrier for APP to catalyze char formation that effectively blocks the propagation of heat and combustible gas generated during the combustion process [42,43,44,45]. EDS maps demonstrate that EP/15RGO-APP char has a higher carbon element content and higher C/O ratio (2.46) compared with EP/15RGO@APP char. The char layer with a high C/O ratio has higher densification and a better thermal barrier effect [46]. The above results show that the char layer formed by EP/RGO-APP has a denser structure and is of better quality.

**Figure 11:**
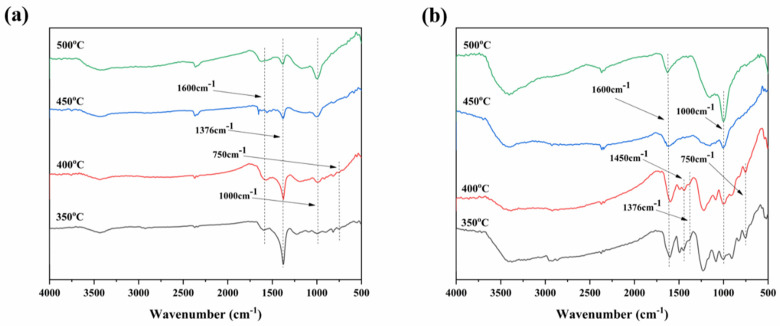
FTIR spectra of the char residues of EP/15RGO@APP (**a**) and EP/15RGO-APP (**b**) heated at different temperatures in a muffle furnace.

In order to further explore the flame-retardant mechanism of RGO-APP and RGO@APP in EP, the FTIR spectra of the EP composites at different temperatures were analyzed, and the results are shown in Figure 11. It can be observed that the FTIR spectra of EP/15RGO@APP and EP/15RGO-APP heated at different temperatures show similar characteristic peaks at 1600 cm^−1^, 1376 cm^−1^, 1000 cm^−1^, and 750 cm^−1^, respectively, which are ascribed to the C=C bond, C=O bond, P-N-C bond, and P-O-C bond, respectively [47,48]. With the increase in temperature, the peaks of the P-N-C bond and P-O-C bond become weaker, while the peaks of the C=C, P-N-C, and P-O-C groups become stronger. This is because of the fact that APP forms acidic compounds such as metaphosphoric acid to catalyze carbonization, resulting in the formation of char precursors including the P-N-C bond and P-O-C bond. Compared with EP/15RGO@APP, the peak intensity of the C=C, P-N-C, and P-O-C groups in EP/15RGO-APP becomes stronger, indicating the formation of more aromatic and cross-linked structures at high temperatures after adding RGO-APP, thus enhancing the stability of the char layer [49]. The results are consistent with the results of a higher carbon content in the EP/15RGO-APP char observed in EDS maps.

The observation of the intensity of infrared spectral peaks reveals the flame-retardant mechanism of RGO-APP. The intensity of the C=C bond and C=O bond of EP/15RGO@APP is stronger than that of RGO-APP, while the intensity of the P-N-C bond and P-O-C bond of RGO@APP is weaker than that of RGO-APP. This is because the agglomeration of RGO@APP leads to the insufficient combustion of EP. Compared with RGO@APP, RGO-APP can be well-dispersed in EP, so as to promote the charring reaction of more acid from EP substrate during combustion, which results in the char residue being dominated by the P-N-C bond and P-O-C bond [50].

### 3.7. Mechanical Test

The tensile strength and elastic modulus of the EP composites are shown in Table 4. The addition of either RGO-APP or RGO@APP greatly enhances the tensile strength and elastic modulus of EP. The enhanced mechanical properties of EP composites are ascribed to the well-dispersed RGO and APP in the EP matrix [51]. In addition, compared with EP/15RGO@APP, EP/15RGO-APP has stronger mechanical properties, which is because the amino group on the surface of RGO-APP increases the curing crosslinking degree of EP and acts as a toughening agent [49]. In general, RGO-APP endows EP composites with superior mechanical properties.

## 4. Conclusions

In this work, a novel intumescent flame retardant named reduced-graphene-oxide-modified ammonium polyphosphate (RGO-APP) was synthesized, and the structure of RGO-APP was characterized by FTIR and SEM. Then, RGO-APP was used to modify EP to prepare flame-retardant EP. The thermal stability, flame retardancy, smoke suppression, and mechanical properties of the flame-retardant EP were analyzed by thermogravimetric analysis, differential scanning calorimetry, limiting oxygen index, vertical combustion grade, cone calorimeter, tensile test, and char layer analysis, and the mechanism of flame retardant was analyzed. The LOI and UL94 tests show that the RGO-APP obtained by the reaction exhibits stronger flame retardancy than RGO@APP, where EP containing 15 wt% of RGO-APP has an LOI value of 35.8% and passes the UL94 V-0 rating. The cone calorimetry analysis shows that RGO-APP has a strong inhibitory effect on the heat release and smoke production of EP. The PHRR and PSPR of EP containing 15 wt% of RGO-APP are decreased by 83.6% and 74.3%, respectively, compared with pure EP. The results of the char layer analysis show that RGO-APP has better dispersibility than RGO@APP, which promotes the formation of a more phosphorus-rich char layer, thereby enhancing the compactness of the char layer formed by EP during pyrolysis. The mechanical properties displayed in the test results show that the good compatibility between the EP matrix and RGO-APP endows EP composites with superior tensile properties. This study provides assistance for the application of a cooperative flame retardant of graphene and ammonium polyphosphate in flame-retardant modified EP.

## Figures and Tables

**Table 1:** Formulations and flammability of the EP composites.

Samples	EP (wt%)	DDM (wt%)	RGO-APP (wt%)	RGO (wt%)	APP (wt%)	LOI (%)	UL94 Rating
EP	80	20	0	0	0	26.3	No rating
EP/5RGO-APP	76	19	5	0	0	26.4	No rating
EP/7.5RGO-APP	74	18.5	7.5	0	0	26.8	No rating
EP/10RGO-APP	72	18	10	0	0	28.3	V-1
EP/12.5RGO-APP	70	17.5	12.5	0	0	32.1	V-1
EP/15RGO-APP	68	17	15	0	0	35.8	V-0
EP/5RGO@APP	76	19	0	4.29	0.71	26.2	No rating
EP/7.5RGO@APP	74	18.5	0	6.43	1.07	26.6	No rating
EP/10RGO@APP	72	18	0	8.57	1.43	27.4	No rating
EP/12.5RGO@APP	70	17.5	0	10.71	1.79	30.8	V-1
EP/15RGO@APP	68	17	0	12.86	2.14	34.1	V-0

**Table 2:** Thermogravimetric analysis and derivative thermogravimetric analysis data of pure EP and flame-retardant EP.

Samples	T_5%_ (°C)	T_max_ (°C)	PMLR (%/min)	W_exp_ (%)
EP	366.9	387.2	−14.7028	21.9
EP/5RGO-APP	335.6	349.3	−18.37081	27.0
EP/10RGO-APP	338.9	352.7	−20.191	28.8
EP/15RGO-APP	339.4	355.4	−22.23751	33.8
EP/15RGO@APP	335.0	352.2	−23.35312	31.4

Abbreviations: T_5%_, the temperature at which the mass loss is 5%; T_max_, the temperature of peak mass loss rate; PMLR, peak mass loss rate; W_exp_, the residual weight of char in the experiment.

**Table 3:** Typical cone test data of pure EP and flame-retardant EP.

Samples	TTI (s)	PHRR (kW/m^2^)	THR (MJ/m^2^)	PSPR (m^2^/s)	TSP (m^2^/m^2^)	Residue (%)
EP	30 ± 2	959.3 ± 25.2	95.8 ± 4.6	0.2536 ± 0.0025	33.4 ± 1.2	1.4 ± 0.2
EP/5RGO-APP	35 ± 2	285.1 ± 10.2	49.4 ± 3.8	0.1561 ± 0.0019	33.7 ± 1.5	10.5 ± 0.7
EP/10RGO-APP	37 ± 3	197.0 ± 8.9	40.4 ± 2.4	0.1186 ± 0.0016	28.6 ± 0.9	12.0 ± 0.6
EP/15RGO-APP	42 ± 3	157.7 ± 6.8	35.8 ± 2.3	0.0653 ± 0.0009	23.8 ± 1.0	13.1 ± 1.1
EP/15GO@APP	39 ± 2	227.7 ± 9.5	40.8 ± 2.5	0.0732 ± 0.0012	28.9 ± 1.1	10.1 ± 0.6

Abbreviations: TTI, time to ignition; PHRR, the peak of heat release rate; THR, total heat release; PSPR, the peak of smoke production rate; TSP, total smoke production.

**Table 4:** Tensile strength and elastic modulus of pure EP and flame-retardant EP.

Samples	Elasticity Modulus (MPa)	Tensile Strength (MPa)
EP	5243.6231	4.341
EP/5RGO-APP	5832.0092	58.764
EP/10RGO-APP	6178.9101	62.344
EP/15RGO-APP	6595.3522	75.102
EP/15RGO@APP	6087.6518	64.337

## Data Availability

The data presented in this study are available on request from the corresponding author.
